# Supplementary material for: Automated food intake tracking requires depth-refined semantic segmentation to rectify visual-volume discordance in long-term care homes
Source: Sci Rep. 2022 Jan 7;12:83. doi: 10.1038/s41598-021-03972-8 (PMC8742067; doi:10.1038/s41598-021-03972-8)
Supplement: Supplementary file 1 — Supplementary Information. [file 41598_2021_3972_MOESM1_ESM.pdf]

Supplementary Materials

S1. Existing Food Database Comparison

Table 1 below provides a comprehensive comparison of existing food databases along with characteristics that are relevant to food intake estimation.

Table 1: Summary of popular food databases and their appropriateness for use in LTC.

Dataset	Content	Portion	Orientation	Acquisition	Label Level	Accessibility	Ref.	LTC Requirements Top	LTC Requirements Pixel	LTC Requirements Intake
PFID	USA Fast Foods	full	variable or angled	Restaurant + lab	image-level	open source	[1]	N	N	N
TADA	USA Foods	n/a	n/a	Lab	n/a	proprietary	[2]	N/A	N/A	N/A
Food85	Japanese Foods	n/a	n/a	Web + prev. dataset	n/a	proprietary	[3]	N/A	N/A	N/A
Chen	Chinese Foods	full	variable or angled	Web	image-level	open source	[4]	N	N	N
UEC Food-100	Japanese Foods	full	variable or angled	Digital camera	bounding box	open source	[5]	N	N	N
UNICT-FD889	Variety of Foods	full	variable or angled	Smart phone	image-level	open source	[6]	N	N	N
Food-101	USA Foods	full	variable or angled	Web	image-level	open source	[7]	N	N	N
UEC Food-256	Japanese & Other Foods	full	variable or angled	Digital camera	bounding box	open source	[8]	N	N	N
Food201-Segmented	USA Foods	full	variable or angled	Web	pixel-level	open source	[9]	N	Y	N
Menu-Match	Restaurant Foods (Asian, Italian, Soup)	full	variable or angled	Digital camera	image-level	open source	[10]	N	N	N
UNIMIB2015	USA Dining Hall	full+ leftovers	top-view	Digital camera	image-level	open source	[11]	Y	N	Y
UNIMIB2016	USA Dining Hall	full	top-view	Digital camera	pixel-level	open source	[11]	Y	Y	N
VireoFood-172	Chinese Foods	full	variable or angled	Web	image-level	open source	[12]	N	N	N
ChineseFoodNet	Chinese Foods	full	variable or angled	Web	image-level	open source	[13]	N	N	N
ChinFood1000	Chinese Foods	full	variable or angled	Web	image-level	open source	[14]	N	N	N
Recipe1M+	USA Foods	full	variable or angled	Web	image-level	open source	[15]	N	N	N
FoodX-251	Variety	full	variable or angled	Web	image-level	open source	[16]	N	N	N
AIFood	Variety	full	variable or angled	Web	image-level	open source	[17]	N	N	N
CROCUFID	Variety	full	variable or angled	Lab	image-level	open source	[18]	N	N	N
FooDD	Entire Foods (e.g., whole apple)	full	variable or angled	Smartphone, Web	pixel-level	open source	[19]	N	Y	N
ISIA Food-500	Variety	full	variable or angled	Web	image-level	open source	[20]	N	N	N

References

[1] Chen, M. *et al.* PFID: Pittsburgh fast-food image dataset. In *2009 16th IEEE International Conference on Image Processing (ICIP)*, 289–292 (IEEE, 2009).

[2] Mariappan, A. *et al.* Personal dietary assessment using mobile devices. In *Computational Imaging VII*, vol. 7246, 72460Z (International Society for Optics and Photonics, 2009).

[3] Hoashi, H., Joutou, T. & Yanai, K. Image recognition of 85 food categories by feature fusion. In *2010 IEEE International Symposium on Multimedia*, 296–301 (IEEE, 2010).

[4] Chen, M.-Y. *et al.* Automatic chinese food identification and quantity estimation. In *SIGGRAPH Asia 2012 Technical Briefs*, 1–4 (2012).

[5] Matsuda, Y., Hoashi, H. & Yanai, K. Recognition of multiple-food images by detecting candidate regions. In *2012 IEEE International Conference on Multimedia and Expo*, 25–30 (IEEE, 2012).

[6] Farinella, G. M., Allegra, D. & Stanco, F. A benchmark dataset to study the representation of food images. In *European Conference on Computer Vision*, 584–599 (Springer, 2014).

[7] Bossard, L., Guillaumin, M. & Van Gool, L. Food-101–mining discriminative components with random forests. In *European conference on computer vision*, 446–461 (Springer, 2014).

[8] Kawano, Y. & Yanai, K. Automatic expansion of a food image dataset leveraging existing categories with domain adaptation. In *European Conference on Computer Vision*, 3–17 (Springer, 2014).

[9] Meyers, A. *et al.* Im2calories: towards an automated mobile vision food diary. In *Proceedings of the IEEE International Conference on Computer Vision*, 1233–1241 (2015).

[10] Beijbom, O., Joshi, N., Morris, D., Saponas, S. & Khullar, S. Menu-match: Restaurant-specific food logging from images. In *2015 IEEE Winter Conference on Applications of Computer Vision*, 844–851 (IEEE, 2015).

[11] Ciocca, G., Napoletano, P. & Schettini, R. Food recognition: a new dataset, experiments and results. *IEEE Journal of Biomedical and Health Informatics* **21**, 588–598 (2017).

[12] Jing-jing Chen, C.-w. N. Deep-based ingredient recognition for cooking recipe retrival. *ACM Multimedia* (2016).

[13] Chen, X., Zhou, H., Zhu, Y. & Diao, L. Chinesefoodnet: A large-scale image dataset for chinese food recognition. *arXiv preprint arXiv:1705.02743* (2017).

[14] Fu, Z., Chen, D. & Li, H. Chinfood1000: A large benchmark dataset for chinese food recognition. In *International Conference on Intelligent Computing*, 273–281 (Springer, 2017).

[15] Marin, J. *et al.* Recipe1m+: A dataset for learning cross-modal embeddings for cooking recipes and food images. *IEEE Trans. Pattern Anal. Mach. Intell.* (2019).

[16] Kaur, P. *et al.* Foodx-251: A dataset for fine-grained food classification. *arXiv preprint arXiv:1907.06167* (2019).

[17] Lee, G. G., Huang, C., Chen, J., Chen, S. & Chen, H. Aifood: A large scale food images dataset for ingredient recognition. In *TENCON 2019 - 2019 IEEE Region 10 Conference (TENCON)*, 802–805 (2019).

[18] Toet, A. *et al.* Crocufid: a cross-cultural food image database for research on food elicited affective responses. *Frontiers in psychology* **10**, 58 (2019).

[19] Parisa Pouladzadeh, S. S., Abdulsalam Yassine. FooDD: Food detection dataset for calorie measurement using food images (2020). URL `https://dx.doi.org/10.21227/yvk7-qk38`.

[20] Min, W. *et al.* *ISIA Food-500: A Dataset for Large-Scale Food Recognition via Stacked Global-Local Attention Network*, 393–401 (Association for Computing Machinery, New York, NY, USA, 2020). URL `https://doi.org/10.1145/3394171.3414031`.
